# Flowers, Ferns, and Ward Decorations

**Published:** 1887-01-01

**Authors:** 


### Flowers, Ferns, and Ward Decorations.

[Continued from page 187.—Communications for this column should be addressed "Gardener," care of the Editor of The Hospital.]

When winter approaches, and the garden can no longer be requisitioned for purposes of indoor decoration, one is glad to fall back upon pot-plants to supply the place of cut flowers. In such places as hospitals, where the regular supply of the latter is at all times uncertain, the sisters and nurses who look beyond the day's needs in this particular, and provide themselves greenery against the dark winter's length are to be commended, as having nothing in common with those foolish virgins whose lamps were found empty at the most inopportune moment!

To insure the welfare of a pot-plant in a London house of any description, the greatest care must be bestowed. Plants, like children, having requirements innumerable; no one who is unendowed with the serpent's wisdom and the patience of long-suffering Job can ever hope to bring either to perfection or to keep them there when brought.

The first thing to be done when a plant is purchased (if it cannot be obtained "in a present," as the Scotch express it) is to see that the pot is a suitable size to hold it. Root-crowding is about as conducive to health in a flower as is toe-squeezing to the growth of a Chinese lady's foot. Do not always be watering your specimens, but never allow the soil to become dry and crumbly. Ferns, above all, must have abundance of moisture. Many of them flourish best standing in a saucer of water; some will not live at all out of a humid atmosphere. Dust is one of the greatest enemies plants have to contend with—they cannot possibly thrive in its presence; and as a satisfactory materfamilias tubs her babies with due regularity, so must a good gardener sponge the leaves of *his* when their surface becomes covered with grime and grit. Too much attention can hardly be paid to this matter. I once asked a cottager how she managed to keep her window-plants so beautifully clean and healthy. "Bless you," was the answer, "I washes 'em as regular as the children"; and judging from the appearance of both, soap and water were well known in that household, and cleanliness occupied its proper place in the order of virtues. In the case of coarse-growing foliage, of course, the sponging can be easily done, a little soft soap in the water being sometimes necessary to kill any of the many pests which infest them. India-rubber plants, aurulias, aucubas, and so on, are always the better for a wash. When plants have soft, fragile foliage, it is wiser to place them outside the window-ledge or on the pavement below-stairs and deluge them gently overhead with a fine rose or syringe till the dust is all gone. Better still, if the plants are left out of doors during the more natural performance of what a local Goody once described as "the heavenly watering-pot." This plan never fails to be successful unless the said vehicle takes to discharging hail and thunder-bolts, accompanied by a tearing blast from the north or east. The finest ferns I ever saw grown in a sitting-room were regularly submitted to the influence of soft summer showers, their owner at the same time keeping a sharp look-out for any intruders among the mould, in the shape of worms, snails, slugs, and garden lice, the three last being especially injurious to ferns, any one of them being capable of supplying the growers with a personal application of the Laureate's "crown of sorrows."

It is impossible to explain the exact amount of water required by each separate variety of growing thing; much must be left to the discretion of the person who undertakes their cultivation. This quality, however, is not always to be counted on, judging from the condition of many London window-boxes. It is to be concluded that the owners thereof share the sentiments of a certain Scotch minister in the Lews, who, on being asked to partake of whiskey-and-water, promptly replied, "A weel, I'll thank ye for a trifle o' the speerit, but I've nae opeenion o' the warther."

The best kinds of roots to be grown in hospitals—and, of course, I do not mean those which are brought in covered with bursting buds, there to blossom and then be removed—are bulbs, ferns, and foliage-plants—big, strong, self-asserting things, like those mentioned above. Some flowers cannot be grown at all where antiseptics are in use, and it is therefore folly to attempt the cultivation of delicate things and stove-house specimens. Much better obtain sensible plants and grow them as near to perfection as possible.

The popular belief that flowers in a room are harmful to invalids is rapidly becoming exploded, though I am still old-fashioned enough to think that "Lincolnshire cauliflowers" and various other strong-smelling lilies are best *outside* the sick-room at night-time. The greatest danger to be incurred from keeping flowers in bedrooms is the possibility of leaving them too long in the same water, which naturally turns thick and offensive, and must be unhealthy. It is also unwholesome to leave dead and decaying foliage and petals among the blossoms. When rooted plants are observed to be turning limp and yellow it is time to examine the roots, and it will be found that green-fly or worms are at work, or the soil has become sour and requires renewing. One of the principal subjects for scrutiny is the drainage of pots; nothing will grow properly if this matter be neglected, because, if choked up, the water cannot properly percolate through, and sticky, mouldy, and foul-smelling soil is the result, wherein no flower can hold its own.

There are many varieties of conifera, green or variegated, which look extremely well as table-plants, many of them delicate, and remarkable for their gold, silver, and glaucous tinting; hardiness being one of their chief recommendations and their cheapness another.

